# Akt-GSK3β-mPTP pathway regulates the mitochondrial dysfunction contributing to odontoblasts apoptosis induced by glucose oxidative stress

**DOI:** 10.1038/s41420-022-00981-y

**Published:** 2022-04-05

**Authors:** Danni Wu, Liya Yan, Chuchu Zheng, Xuekun Ren, Yihuai Pan, Shengbin Huang, Lijun Pan, Zongli Li

**Affiliations:** 1 Institute of Stomatology, School and Hospital of Stomatology, Wenzhou Medical University, Wenzhou, 325027 China; 2 Department of Endodontics, School and Hospital of Stomatology, Wenzhou Medical University, Wenzhou, 325027 China; 3 Department of Prosthodontics, School and Hospital of Stomatology, Wenzhou Medical University, Wenzhou, 325027 China

**Keywords:** Apoptosis, Oral diseases

## Abstract

Diabetes Mellitus can cause dental pulp cells apoptosis by oxidative stress, and affect the integrity and function of dental pulp tissue. Mitochondria are the main attack targets of oxidative stress and have a critical role in apoptosis. However, whether mitochondria are involved in dental pulp damage caused by diabetes mellitus remains unclear. This study aimed to investigate the role of mitochondria in the apoptosis of odontoblast-like cell line (mDPC6T) induced by glucose oxidative stress, and to explore its possible mechanism. We established an oxidative stress model in vitro using glucose oxidase/glucose to simulate the pathological state under diabetic conditions. We found that the opening of mitochondrial permeability transition pore (mPTP) contributed to the apoptosis of mDPC6T treated with glucose oxidase, as evidenced by enhanced mitochondrial reactive oxygen species (mtROS) and intracellular Ca^2+^ disorder, significantly reduced mitochondrial membrane potential (MMP) and ATP production. Antioxidant N-acetylcysteine (NAC) or Cyclosporine A (mPTP inhibitor) blocked the mPTP opening, which significantly attenuated mitochondrial dysfunction and apoptosis induced by glucose oxidative stress. In addition, we found that glucose oxidative stress stimulated mPTP opening may through inhibition of Akt-GSK3β pathway. This study provides a new insight into the mitochondrial mechanism underlying diabetes-associated odontoblast-like cell apoptosis, laying a foundation for the prevention and treatment of diabetes-associated pulp injury.

## Introduction

Dental pulp, surrounded by hard and inelastic dentin, is the only loose connective tissue of tooth. It has microcirculatory system only through tiny apical foramen with no collateral circulation. Therefore, the dental pulp is highly sensitive to various external stimuli and prone to irreversible pulp injury, leading to the pathological state of pulp [1–5]. Diabetes Mellitus (DM) characterized by hyperglycemia is a systemic metabolic disease, which caused by insufficient insulin secretion and/or insulin resistance [6]. DM can affect the integrity and function of dental pulp [7, 8], mainly manifested in extensive collagen fibrosis, reduction of blood vessels, increased vasculopathy [9], and higher possibility of calcification [2]. A retrospective study has shown that DM can increase pulp sensitivity, resulting in higher prevalence of symptomatic irreversible pulpitis [10] and exacerbate apical periodontitis [11]. Patients with poorly controlled blood glucose have a low response to endodontic treatment [12] and a high risk of tooth extraction after nonsurgical root canal treatment [13]. Animal models have demonstrated that diabetic mice were more vulnerable to dental caries [14], dental pulp necrosis and apical periodontitis [15]. It has also been indicated that high glucose inhibited proliferation and induced apoptosis of human dental pulp cells in vitro [16]. Therefore, based on the basic research and clinical evidence, molecular knowledge of diabetes-associated dental pulp cell damage is essential for finding the potential preventive/therapeutic strategies for the endodontic disease of diabetic patients. However, the mechanism underlying diabetes-associated dental pulp injury remains largely unknown.

Hyperglycemia exacerbates glucose oxidation and mediates excessive production of reactive oxygen species (ROS), leading to oxidative stress and irreversible damage of dental pulp cells, which has been identified as the main mechanism of dental pulp injury caused by hyperglycemia [17]. Mitochondria are the main source and the primary attacking target of ROS [18]. Excessive ROS can cause rapid consumption of antioxidants, which results in oxidative stress and mitochondria dysfunction in human dental pulp cells (HDPCs) [19]. The application of mitochondria specific antioxidants can inhibit mitochondrial dysfunctions of HDPCs and alleviate pulpitis [20]. All these results suggest that the mitochondrial oxidative damage may be the cause of the dental pulp injury by hyperglycemia.

Protein kinase B (Akt), a serine-threonine protein kinase, is a critical regulator of cell proliferation and survival. Lipoteichoic acid and Dycal co-treatment induced dental pulp stem cells apoptosis by increasing level of Akt and decreasing the level of phosphorylated Akt [21]. Adrenomedullin inhibited apoptosis of dental pulp stem cells by activating glycogen synthase kinase 3β (GSK3β) pathway [22]. Activated Akt inhibits glycogen synthase kinase 3β (GSK3β) by increasing its phosphorylation. Moreover, inactivation of Akt-GSK3β resulted in mitochondrial dysfunction and apoptosis in osteoblast induced by hydrogen peroxide (H_2_O_2_) [23]. Whether Akt-GSK3β is involved in diabetes-associated dental pulp injury remains to be elucidated.

The mitochondrial permeability transition pore (mPTP), as a nonspecific channel located between the inner and outer membrane of mitochondria, is formed by conformational changes of mitochondrial membrane proteins [24]. Under conditions of ROS overload, mPTP is activated and opened, leads to mitochondrial dysfunction-associated damage and apoptosis [25, 26]. Akt-GSK3β is closely involved in mPTP regulation. A decreased phosphorylated GSK3β was shown to less interact with ANT, thus facilitate the formation of CypD-ANT complex, known as components of mPTP, which was suggested to be responsible for the promotion of mPTP opening and mitochondrial dysfunction [27]. Inhibition of GSK3β protects against mitochondrial dysfunction and sensitizing the mPTP in triptolide-induced acute cardiac injury [28]. Meanwhile, inhibition of GSK3β activity can protect the function of mitochondria and alleviate cognitive decline in diabetic mice [29, 30]. Therefore, we hypothesized that glucose oxidase-induced mPTP opening, leading to mitochondrial dysfunction and apoptosis by decreasing phosphorylation of Akt-GSK3β signaling.

On the whole, the purpose of the present study was to investigate (1) the role of mPTP in dental pulp cells injury induced by glucose oxidative stress; (2) the relationship between the Akt-GSK3β signaling pathway and mPTP in this process.

## Results

### Glucose oxidase impaired cell viability and induced apoptosis in mDPC6T cells

**Fig. 1:**
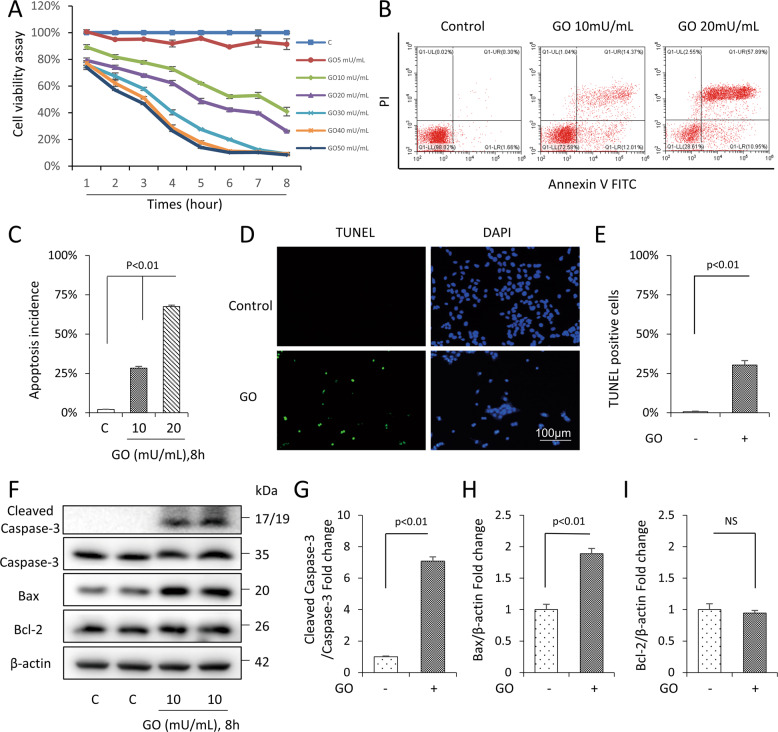
Glucose oxidase-induced apoptosis in mDPC6T cells. **A** The cell viability was assessed by CCK8 assay. **B**, **C** Flow cytometric quantification of apoptosis. **D**, **E** TUNEL assay was performed to determine the rate of cell apoptosis. **F**–**I** The expressions of apoptotic proteins (including cleaved Caspase-3, Caspase-3, Bax, Bcl2) in mDPC6T cells in the presence of glucose oxidase. The values are expressed as the means ± SD (*n* = 3).

The mDPC6T cells were incubated with 5-50 mU/mL glucose oxidase for 1–8 h. Glucose oxidase treatment significantly reduced the number of viable cells in a time- and dose-dependent manner (Fig. 1A). The median lethal dose of glucose oxidase was 10 mU/mL after 8 h treatment. The results of flow cytometric showed a dose-dependent increase in the incidence of cell apoptosis (Fig. 1B, C). Glucose oxidase-induced DNA damage was further confirmed by a significantly increased TUNEL-positive staining (Fig. 1D, E). Furthermore, compared with the control, glucose oxidase significantly enhanced the expression of apoptotic protein cleaved-Caspase3 and Bax, while no significant differences were observed in antiapoptotic protein Bcl-2 expression (Fig. 1F–I), which was consistent with our previous findings in H_2_O_2_-induced oxidative stress cell model [31]. These results indicated that glucose oxidase-induced cell death occurred primarily through apoptosis in mDPC6T cells.

### Glucose oxidase treatment resulted in mitochondrial dysfunction and exacerbated mPTP opening in mDPC6T cells

**Fig. 2:**
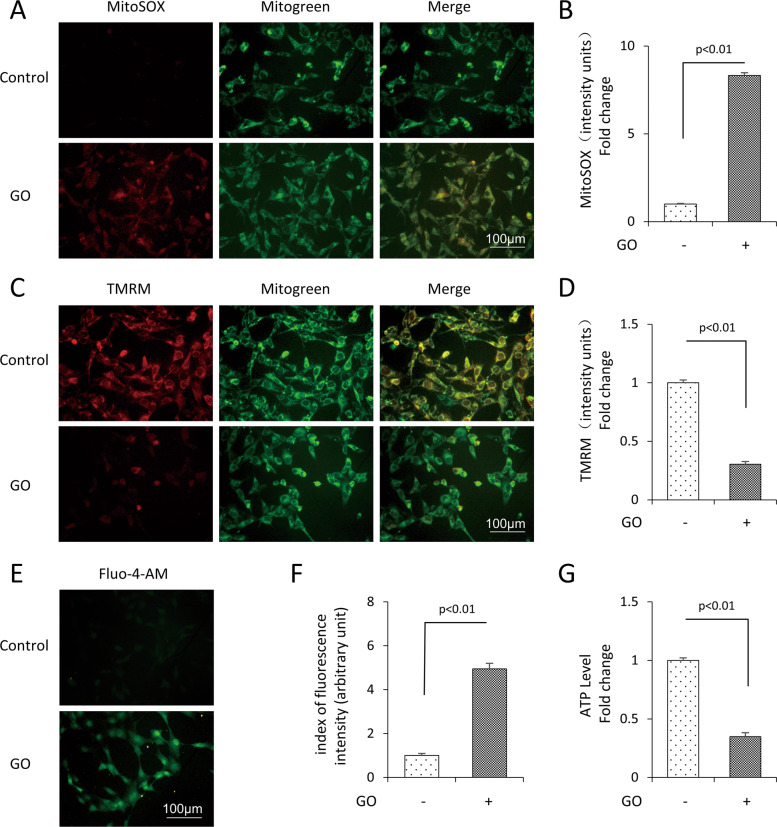
Glucose oxidase treatment induced mitochondrial oxidative damage in mDPC6T cells. MitoSOX staining (**A**) and quantification (**B**) in the indicated groups. TMRM staining (**C**) and quantification (**D**) in the indicated groups. Fluo-4-AM staining (**E**) and quantification (**F**) in the indicated groups. **G** ATP in the indicated groups. The values are expressed as the means ± SD (*n* = 3).

To attest mitochondrial oxidative damage in glucose oxidative stress, we detected mitochondrial function. We found that glucose oxidase significantly increased the level of mtROS (Fig. 2A, B), suppressed MMP (Fig. 2C, D) and reduced ATP production (Fig. 2G). Furthermore, glucose oxidase markedly increased the intracellular Ca^2+^ level (Fig. 2E, F). These results showed that glucose oxidase treatment resulted in mitochondrial dysfunction, meanwhile suggested that the opening of mPTP may participate in the process.

**Fig. 3:**
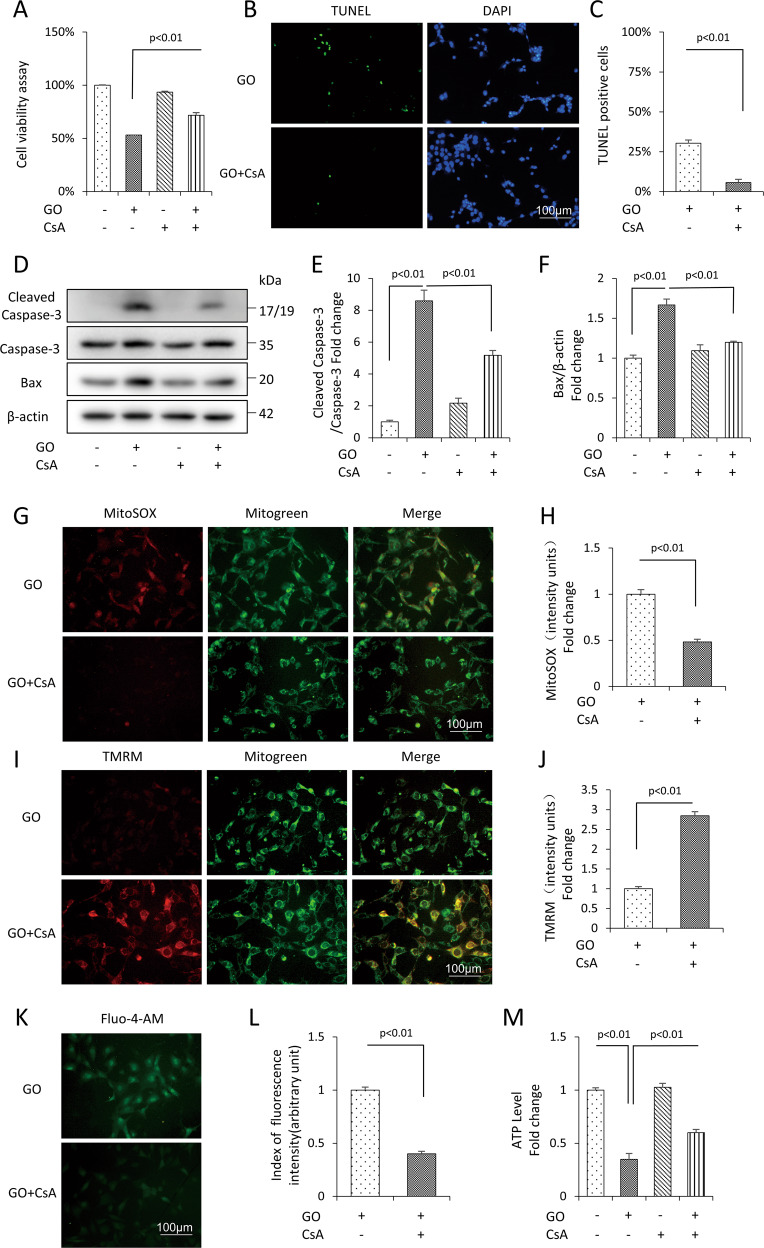
Inhibition of mPTP by CsA reversed glucose oxidase-induced apoptosis and mitochondrial oxidative damage in mDPC6T cells. **A** Cell viability determined by CCK8 assay in the presence of glucose oxidase with or without CsA. **B**, **C** TUNEL staining and assay. **D**–**F** Protein level of cleaved Caspase-3, Bax by Western blot and quantification analysis. MitoSOX staining (**G**) and quantification (**H**) in the indicated groups. TMRM staining (**I**) and quantification (**J**) in the indicated groups. Fluo-4-AM staining (**K**) and quantification (**L**) in the indicated groups. **M** ATP in the indicated groups. The values are expressed as the means ± SD (*n* = 3).

To further delineate the precise role of mPTP in glucose oxidative stress, we incubated mDPC6T cells with mPTP inhibitor CsA. CCK8 assay showed that CsA treatment rescued cell viability (Fig. 3A). These results were further confirmed by TUNEL staining (Fig. 3B, C). CsA also significantly downregulated the expression level of cleaved Caspase-3 and Bax (Fig. 3D–F). Furthermore, CsA significantly alleviated the level of mtROS generation (Fig. 3G, H) and the intracellular Ca^2+^ level (Fig. 3K, L). CsA also enhanced the MMP (Fig. 3I, J) and ATP level (Fig. 3M). These results showed glucose oxidase-induced mPTP opening resulted in apoptosis and mitochondrial dysfunction, which can be reversed by treatment with the mPTP opening inhibitor CsA.

### NAC alleviated glucose oxidase-induced mitochondrial dysfunction and apoptosis of mDPC6T cells

**Fig. 4:**
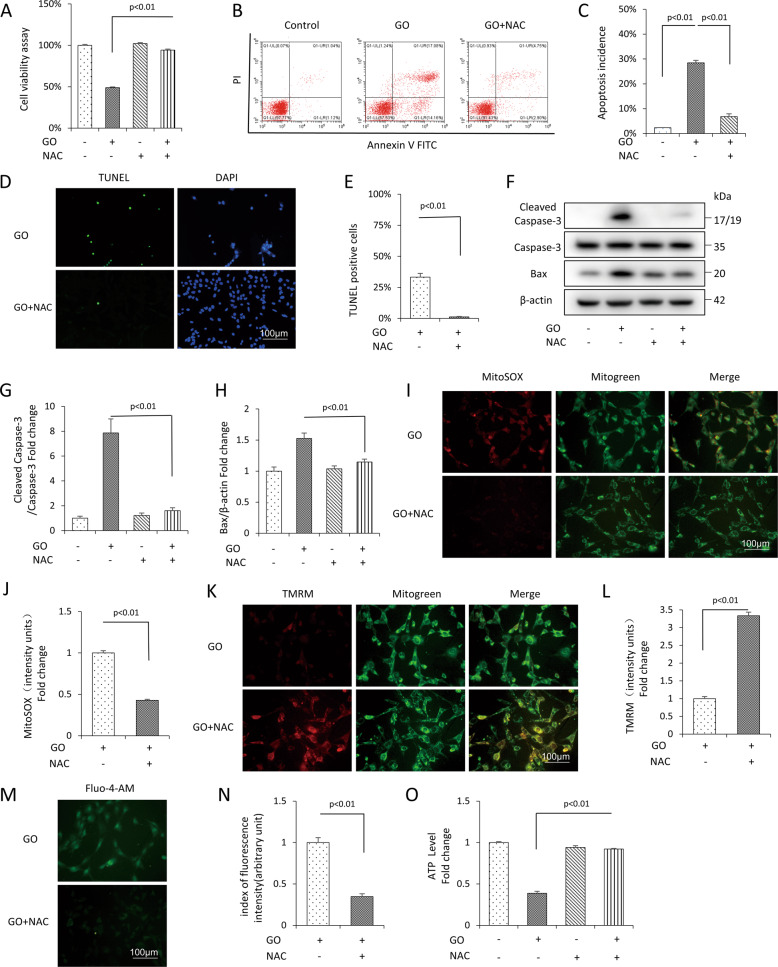
The antioxidant NAC attenuated glucose oxidase-induced apoptosis and mitochondrial oxidative damage in mDPC6T cells. **A** Cell viability determined by CCK8 assay in the presence of glucose oxidase with or without NAC. **B**, **C** Flow cytometric quantification of apoptosis. **D**, **E** TUNEL staining and assay after NAC treatment. **F**–**H** Protein level of cleaved Caspase-3, Bax by Western blot and quantification analysis. MitoSOX staining (**I**) and quantification (**J**) in the indicated groups. TMRM staining (**K**) and quantification (**L**) in the indicated groups. Fluo-4-AM staining (**M**) and quantification (**N**) in the indicated groups. **O** ATP in the indicated groups. The values are expressed as the means ± SD (*n* = 3).

To further identify whether glucose oxidase stress-induced mitochondrial dysfunction and apoptosis, we used NAC, a nonspecific antioxidant, in the presence of glucose oxidase. We observed NAC significantly prevented the cell viability (Fig. 4A) and decreased apoptosis compared with the cells treated with glucose oxidase alone (Fig. 4B–E). The protein expression of apoptotic protein cleaved Caspase-3 and Bax significantly decreased (Fig. 4F–H). In addition, we found that NAC protected the mitochondrial function from significantly abrogating the level of mtROS generation (Fig. 4I, J) and the intracellular Ca^2+^ level (Fig. 4M, N), increased MMP (Fig. 4K, L) and ATP level (Fig. 4O).

### Glucose oxidase-induced apoptosis via a Akt-GSK3β signaling pathway

**Fig. 5:**
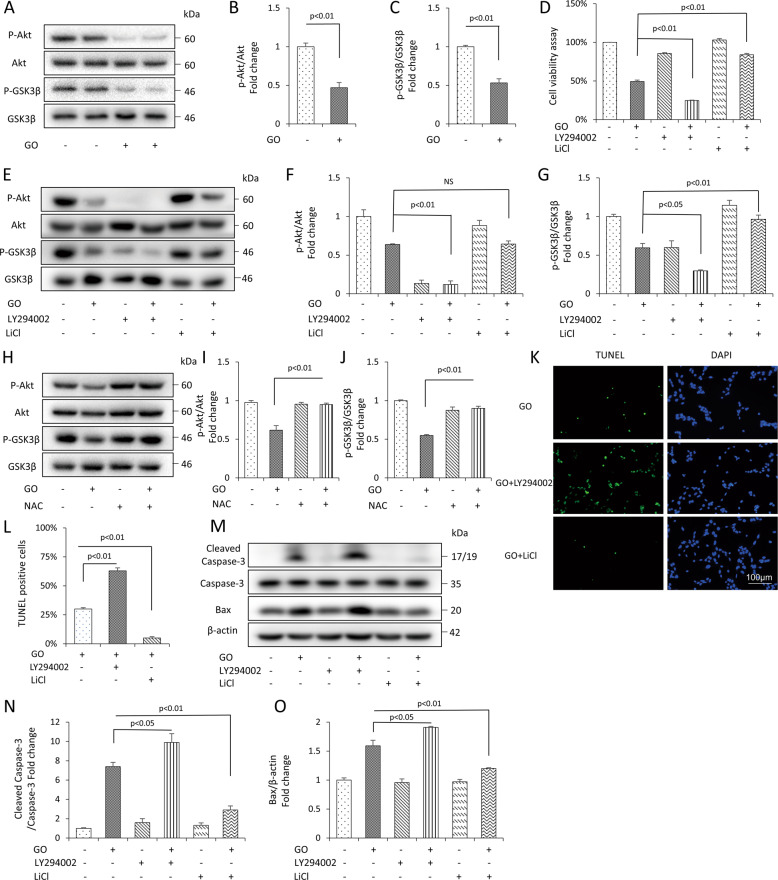
Involvement of Akt-GSK3β signaling pathway in glucose oxidase-induced apoptosis in the mDPC6T cells. **A**–**C** Protein level of p-Akt and Akt, p-GSK3β and GSK3β in the presence of glucose oxidase by Western blot and quantification analysis. **D** The cell viability was assessed by CCK8 assays treated with (+) or without (−) LY294002 or LiCl in the presence (+) or absence (−) of glucose oxidase. **E**–**G** Protein level of p-Akt and Akt, p-GSK3β and GSK3β treated with (+) or without (−) LY294002 or LiCl in the presence (+) or absence (−) of glucose oxidase by Western blot and quantification analysis. **H**–**J** Protein level of p-Akt and Akt, p-GSK3β and GSK3β treated with (+) or without (−) NAC in the presence (+) or absence (−) of glucose oxidase by Western blot and quantification analysis. **K**, **L** TUNEL staining and assay. **M**–**O** Protein level of cleaved Caspase-3, Bax treated with (+) or without (−) LY294002 or LiCl in the presence (+) or absence (−) of glucose oxidase by Western blot and quantification analysis. The values are expressed as the means ± SD (*n* = 3).

Our previous study has reported that the Akt-GSK3β signaling pathway is involved in oxidative stress-induced osteoblast apoptosis [32]. Interestingly, in the present study, glucose oxidase treatment could dramatically reduce the level of p-Akt and p-GSK3β (Fig. 5A–C), without any changes in total Akt and GSK3β, suggesting the potential role of Akt-GSK3β signaling pathway in glucose oxidase-induced apoptosis of mDPC6T cells.

To further assess whether Akt-GSK3β was involved in regulating apoptosis under glucose oxidase condition, we employed LY294002 (PI3K inhibitor) or LiCl (GSK3β inhibitor), followed by glucose oxidase treatment. As shown in (Fig. 5E–G), LY294002 blocked the expression of p-Akt and p-GSK3β. Besides, LY294002 inhibited cell viability and exacerbated glucose oxidase-induced apoptosis, as confirmed by CCK8 (Fig. 5D), TUNEL staining (Fig. 5K, L). LY294002 also promoted the apoptosis protein expression of cleaved Caspase-3 and Bax (Fig. 5M–O). LiCl upregulated the expression of p-GSK3β, but did not affect the restoration of p-Akt (Fig. 5E–G). The results verified the hypothesis that Akt regulates GSK3β phosphorylation. Moreover, LiCl prevented the cell viability and attenuated apoptosis, which was consistent with the results of CCK8 (Fig. 5D) and TUNEL staining (Fig. 5K, L). LiCl also downregulated the protein expression of cleaved Caspase-3 and Bax (Fig. 5M–O). The addition of NAC exerted a protective effect by increasing the level of p-Akt and p-GSK3β (Fig. 5H–J). These results suggested that Akt-GSK3β signaling pathway regulated glucose oxidative stress-induced apoptosis.

### Glucose oxidase activated mPTP opening through the Akt-GSK3β signaling pathway

**Fig. 6:**
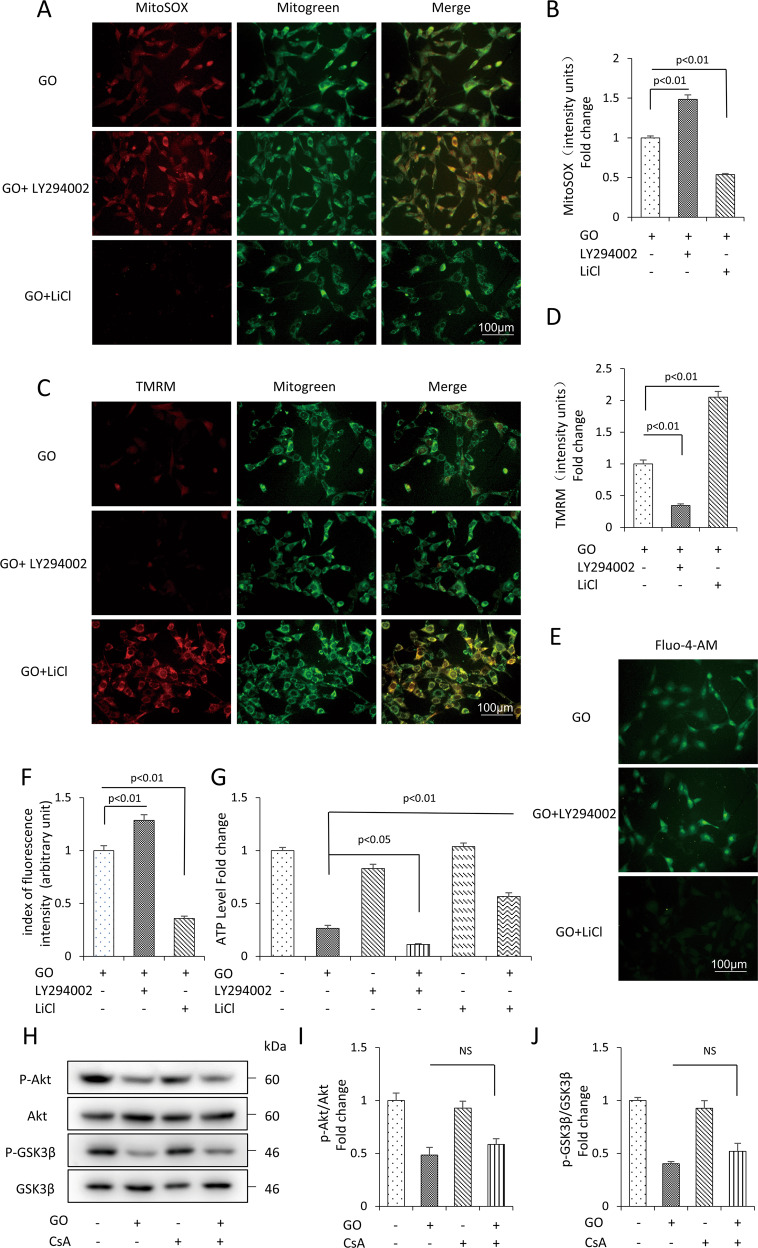
Glucose oxidative acted through the Akt-GSK3β pathway to induce mPTP opening in mDPC6T cells. MitoSOX staining (**A**) and quantification (**B**) in the indicated groups. TMRM staining (**C**) and quantification (**D**) in the indicated groups. Fluo-4-AM staining (**E**) and quantification (**F**) in the indicated groups. **G** ATP in the indicated groups. **H**–**J** Protein level of p-Akt and Akt, p-GSK3β and GSK3β treated with (+) or without (−) CsA in the presence (+) or absence (−) of glucose oxidase by Western blot and quantification analysis. The values are expressed as the means ± SD (*n* = 3).

Considering Akt-GSK3β signaling was known to function as a pro-survival signal, we hypothesized that glucose oxidase activating mPTP opening might be due to the inhibition of Akt-GSK3β signaling pathway. We measured the function of mitochondria treated with LY294002 or LiCl in the presence of glucose oxidase. As expected, LY294002 aggravated mitochondrial damage induced by glucose oxidase, which was reflected in increased mtROS (Fig. 6A, B) and intracellular Ca^2+^ level (Fig. 6E, F), decreased MMP (Fig. 6C, D) and ATP level (Fig. 6G). Furthermore, these mitochondrial damage indexes were attenuated by LiCl co-treatment with glucose oxidase. LiCl alleviated mtROS level (Fig. 6A, B) and intracellular Ca^2+^ level (Fig. 6E, F), increased the MMP (Fig. 6C, D) and ATP level (Fig. 6G). Additionally, we tested the effect of CsA on the expression of p-Akt and p-GSK3β. As expected, CsA inhibited the opening of mPTP without affecting the expression of p-Akt and p-GSK3β (Fig. 6H–J). These results provided direct evidence suggesting that mPTP served as a downstream process of Akt-GSK3β pathway.

## Discussion

Accumulating evidences have indicated that DM induced oxidative damage to pulp tissue, with impaired pulp cell viability and defensive response of dentin-pulp complex [33]. However, the molecular mechanisms responsible for dental pulp injury in diabetic patients are poorly understood. This study attempted to reveal the pathological mechanism of dental pulp cells injury by diabetic hyperglycemia from the perspective of mitochondrial molecular biology. Our main findings indicated the key role of mPTP in the glucose oxidative stress-induced mitochondrial apoptosis of mDPC6T cells. Blockage mPTP opening could attenuate the oxidative damage to mDPC6T cells. Furthermore, we found Akt-GSK3β regulated mPTP opening. Our results may contribute to identifying the mechanisms of DM induced dental pulp damage and providing a novel therapeutic target to resist the adverse effects of hyperglycemia.

Oxidative stress is a common pathological state of DM and plays an important role in the progression of DM complications [34]. Dental pulp, given its sensitivity and vulnerability, is considered a major target of oxidative stress [35]. It was reported that glucose oxidase could produce H_2_O_2_ slowly and continuously under high glucose condition, which leaded to oxidative stress in dental pulp cells [17, 33]. Therefore, in the present study, we used high glucose medium with glucose oxidase to establish an in vitro cell model of glucose oxidative stress in mDPC6T. Different from the above reported 5 mU/mL, the dose of glucose oxidase was 10 mU/mL in our study. Differences in experimental conditions and cell types may be accountable for the discrepancies in dosage of glucose oxidase.

Apoptosis is one of the main manifestations of tissue damage mediated by the pathological state of DM. Yan et al. showed that high glucose suppressed the proliferation, induced the apoptosis and inhibited the differentiation of human dental pulp cells [16]. Consistent with Yan et al. study, we found that glucose oxidase treatment decreased cell viability and significantly increased the apoptosis of the mDPC6T cells, as indicated by the results of flow cytometry, TUNEL assays, and Western blot. In addition, the antioxidant NAC attenuated oxidative stress and significantly reduced apoptosis. These results confirmed the critical role of glucose oxidative stress in glucose oxidase-induced apoptosis of mDPC6T cells.

Mitochondria are important organelles for cell energy metabolism and maintenance of redox balance, and are also involved in apoptosis signals [36]. mPTP opening is a key prerequisite for inducing mitochondrial-mediated apoptosis [25, 37]. Recent studies have shown that mPTP plays a critical role in tubular epithelial cell and spinal cord cell injury by high glucose [38, 39]. Therefore, we hypothesized that mPTP may be the pivotal cause of glucose oxidative stress-induced mDPC6T cells apoptosis. mPTP activated by reactive oxygen species (ROS) and matrix calcium overloading, and manipulated additionally by a number of associated proteins and the post-translational modifications and binding of ions to these proteins and to the channel itself [40]. The opening of mPTP leads to MPP rapid loss, mitochondrial membrane permeability destruction, mitochondrial Ca^2+^ efflux, mitochondrial swelling and biological energy depletion. In the current study, glucose oxidase treatment facilitated the opening of mPTP, as reflected by mtROS increase, MMP dissipation, Ca^2+^ disorder, and ATP decline. Besides, NAC successfully inhibited mPTP opening and rescued mitochondrial dysfunction, thereby attenuating mDPC6T apoptosis. The protective effect of NAC was also demonstrated in glucose oxidase-treated H9c2 cells [41]. Using CsA, a selective mPTP opening inhibitor, can protect mitochondria and reduce rat kidney proximal tubular cells (NRK-52E) apoptosis induced by high glucose [38]. As expected, our results showed that CsA significantly reversed mitochondrial dysfunction induced by glucose oxidative stress and prevented apoptosis. Taken together, it indicated that mPTP opening played a regulatory role in the apoptosis of mDPC6T cells induced by glucose oxidative stress.

The biomolecular configuration of the mPTP complex has long been debated. It is generally believed mainly composed of adenine nucleotide translocase (ANT), mitochondrial phosphate carrier (PIC), metalloprotease spastic paraplegia 7 (SPG7), the voltage-dependent anion channel (VDAC), and Bcl-2 family members. Besides, CypD is recognized as a real regulator of mPTP but it is not a structural pore component [42]. Our previous studies have shown that upregulated the CypD protein level facilitated to mPTP opening and caused mitochondrial dysfunction, leading to cell death of HDPCs [31]. Therefore, it would be highly interesting to explore whether these components of mPTP complex are associated with mitochondrial oxidative damage during glucose oxidative stress.

Akt-GSK3β signaling pathway has been confirmed to affect mitochondrial redox homeostasis and apoptosis [32]. Thus, we investigated whether this signaling pathway was involved in the apoptosis of mDPC6T cells induced by glucose oxidative stress. Our results showed notable suppression of Akt and GSK3β phosphorylation by glucose oxidase treatment. However, it remained to investigate whether Akt-GSK3β participated in the regulation of mPTP in mitochondrial apoptosis. Phosphorylation of GSK3β can interact with ANT to weaken the formation of ANT-CypD complex, thereby inhibiting the opening of mPTP and reducing myocardial cell apoptosis after ischemia-reperfusion injury [28]. Akt-GSK3β reduced VDAC1 phosphorylation, inhibiting VDAC1 to Bax binding and promoting VDAC1 to HK2 binding, thereby protecting cardiomyocytes against anoxia/reoxygenation-induced injury [43]. In the current study, Akt inhibitor promoted mPTP opening and aggravated mitochondrial dysfunction, whereas GSK3β inhibitor rescued them. Besides, inhibition of mPTP opening by CsA restored mitochondrial function without affecting Akt and GSK3β phosphorylation. These results further confirmed Akt-GSK3β acts as the upstream regulator of mPTP, which was consistent with the previous findings in mice heart ischemia-reperfusion injury [44]. Thus, we may conclude that Akt-GSK3β signaling pathway is involved in glucose oxidative stress-induced apoptosis by regulating the opening of mPTP. However, the exact interaction between Akt-GSK3β and the components of mPTP needs to be further explored.

**Fig. 7:**
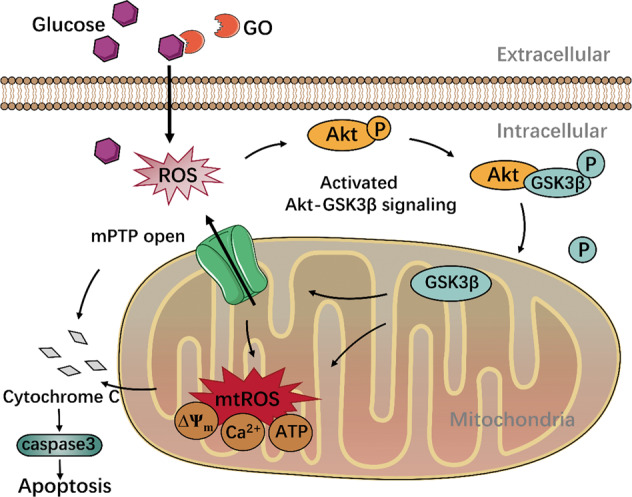
Schematic diagram of the proposed mechanism. Glucose oxidase-induced mitochondrial oxidative damage via the Akt-GSK3β-mPTP signaling pathway to exacerbate mDPC6T cells from apoptosis.

In conclusion, our study demonstrated that glucose oxidative stress exacerbated mPTP opening through inhibited Akt-GSK3β pathway, thereby inducing mDPC6T cells apoptosis (Fig. 7). These findings contribute to our understanding of the pathological mechanism of diabetes-associated dental pulp injury and may have implications for clinical treatment. However, further in-vivo studies should be performed to prove the effect of mPTP in glucose oxidative stress-induced apoptosis and corroborate the molecular mechanisms found in this study.

## Materials and methods

### Reagents

Dulbecco’s Modified Eagle Medium (DMEM) (#11995065), Fetal bovine serum (FBS) (#16000-044), MitoSOX Red (#M36008), Mitogreen (#M7514), and TMRM (#T668) were purchased from Life Technologies (Grand Island, NY, USA). TUNEL assay kit was from Roche (Mannheim, Germany). Annexin V-fluorescein isothiocyanate (FITC) apoptosis detection kit was obtained from BD Bioscience (NJ, USA). Fluo-4-AM, ATP assay kit were obtained from Beyotime Institute of Biotechnology (Shanghai, China). Glucose oxidase (GO) (#G2133), N-acetylcysteine (NAC) (#A7250), Cyclosporine A (CsA) (#SML1018) and Lithium chloride (LiCl) (#L9650) were obtained from Sigma-Aldrich (St. Louis, MO, USA). LY294002 (#9901) was from Cell Signaling Technology (Beverly, MA, USA). Antibodies against phosphorylated Akt (Ser473) (p-Akt) (#4060 S), Akt (#2920 S), phosphorylated GSK3β (Ser9) (p-GSK3β) (#9336 S), GSK3β (#9832 S), Bax (#2772 S), Bcl2 (#3498 S), Caspase-3 (#9662 S), and β-actin (#3700) were obtained from Cell Signaling Technology (Beverly, MA, USA), and antibody CypD (ab110324) was obtained from Abcam (USA). Chamber slides, goat anti-rabbit (#656120) and anti-mouse (#626520) secondary antibodies, and DAPI (#P36931) were obtained from Invitrogen (Carlsbad, CA, USA)

### Cell culture

mDPC6T cells, a pre-odontoblast cell line, were obtained from the School and Hospital of Stomatology, Wuhan University (Wuhan, China). Cells were cultured in DMEM supplemented with 10% FBS and 1% antibiotics (100 ng/ml streptomycin and 100 IU/ml penicillin G) at 37 °C in a humidified incubator with 5% CO_2_. All cell passage numbers used in this study were less than ten passages.

### Cell treatment

Glucose oxidase was dissolved in sodium acetate buffer (PH = 5.5), NAC and LiCl stock solutions were prepared in deionized water. LY294002 and CsA were dissolved in dimethyl sulfoxide (DMSO). The final concentration of DMSO in the culture was less than 0.5%. Stock solutions were diluted to the desired concentration directly in the DMEM before use. The working concentrations of the compounds were as follows: glucose oxidase (10 mU/ml), NAC(2.5 μM), CsA (2 μM), LY294002 (10 μM), and LiCl (10 mM). Cells were treated with or without glucose oxidase and the indicated test compounds for various times, according to the experimental protocol.

### Cell viability assay

mDPC6T cells were seeded in 96-well plates at 1 × 10^4^ cells/well and treated with the conditions as experiment design. The cell viability was then measured with a CCK-8 assay kit according to the manufacturer’s instructions.

### Measurement of apoptosis by flow cytometry and TUNEL assays

mDPC6T cells were seeded in 6-well plates at 3 × 10^5^ cells/well and treated with the conditions as experiment design. Following treatment, cells were digested with 0.25% trypsin without EDTA, washed with PBS, then stained with Annexin V-FITC/PI for 20 min. The apoptosis rates of cells were analyzed using a CytExpert (Carlsbad, CA, USA). For the TUNEL assays, mDPC6T cells were plated in a coverslip, treated with the indicated reagents and fixed in 4% PFA (para-formaldehyde) in PBS and permeabilized with 0.2% Triton X-100 in citrate buffer. Next, the cells were incubated with TUNEL reaction mixture at 37 °C for 1 h, counterstained with DAPI for 10 min. The percentage of apoptotic cells was estimated by counting a total of 300 cells from random fields.

### Western blot analyses

After the indicated treatments, mDPC6T cells were collected and lysed in cell lysis buffer (Cell Signaling Technology, Beverly, MA, USA). Proteins were separated using SDS-PAGE, followed by electrophoretic transfer onto a PVDF membrane. The primary antibody was added and the membrane was incubated overnight at 4 °C, the secondary antibodies were added at room temperature for 1 h. Then using an enhanced chemiluminescence substrate (Thermo Fisher Scientific), images were visualized by the Bio-Rad imaging system (Bio-Rad, Hercules, CA, USA). Densitometry quantification of protein bands were performed with NIH Image J software (Bethesda, MA, USA).

### MtROS and MMP determination

Cells were seeded in 48-well plates at a density of 3 × 10^4^ cells/well and treated with the indicated reagents. To assess the mitochondrial reactive oxygen species (mtROS), we used the combined staining with 2 μM MitoSOX and 100 nM Mitogreen for 30 min in the dark. To assess the mitochondrial membrane potential (MMP), we used the combined staining with 100 nM TMRM and 100 nM Mitogreen for 30 min in the dark. Images were captured under a fluorescence microscope. The quantification and measurement of fluorescent signals were performed with NIH Image J software. Each experiment were performed in triplicate. The fluorescent intensity of more than 100 clearly identifiable mitochondrial in randomly selected 10–15 cells per experiment were measured by an investigator who blinded to experimental design.

### Measurement of ATP level

Cells were collected by lysis buffer provided in the ATP assay kit, then centrifuged and transferred to eppendorf tubes for the analysis of ATP production. Next, the supernatant was mixed with ATP detection solution, and read by luminometry with a microplate reader.

### Detection of Ca^2+^ level

Cells were seeded in 48-well plates at a density of 3 × 10^4^ cells/well and treated with the indicated reagents. To assess the Ca^2+^ Level, we used the combined staining with 2.5 μM Fluo-4-AM for 30 min in the dark. Then, the Ca^2+^ level was detected under the fluorescence microscope.

### Data analysis

Data were shown as mean ± SD and considered significant at *p* < 0.05. All assays were at least performed in triplicate independently. Statistically significant differences were evaluated by Student’s *t*-test (for two groups) and one-way ANOVA (for multi-group). Statistical analysis was performed by StatView software (version 5.0.1, SAS Institute, USA).

## Supplementary information

full western blots

## Data Availability

The data used to support the findings of this study are available from the corresponding authors upon request.
