# Supplementary material for: Akt-GSK3β-mPTP pathway regulates the mitochondrial dysfunction contributing to odontoblasts apoptosis induced by glucose oxidative stress
Source: Cell Death Discov. 2022 Apr 5;8:168. doi: 10.1038/s41420-022-00981-y (PMC8983683; doi:10.1038/s41420-022-00981-y)
Supplement: Supplementary file 1 — full western blots [file 41420_2022_981_MOESM1_ESM.pdf]

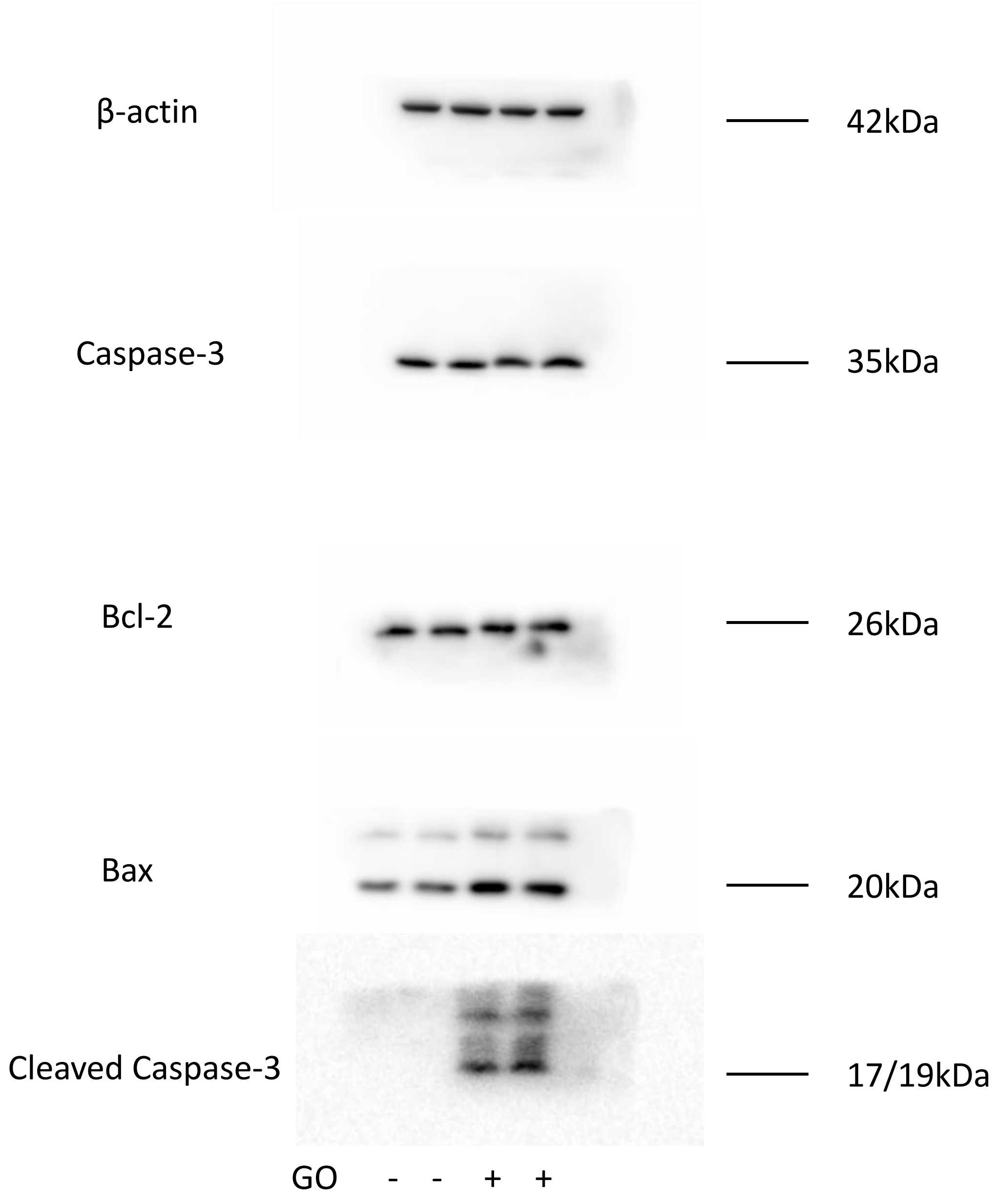

Figure. 1.
(F) The expressions of apoptotic proteins (including cleaved Caspase-3, Caspase-3, Bax, Bcl2) in mDPC6T cells in the presence of glucose oxidase.

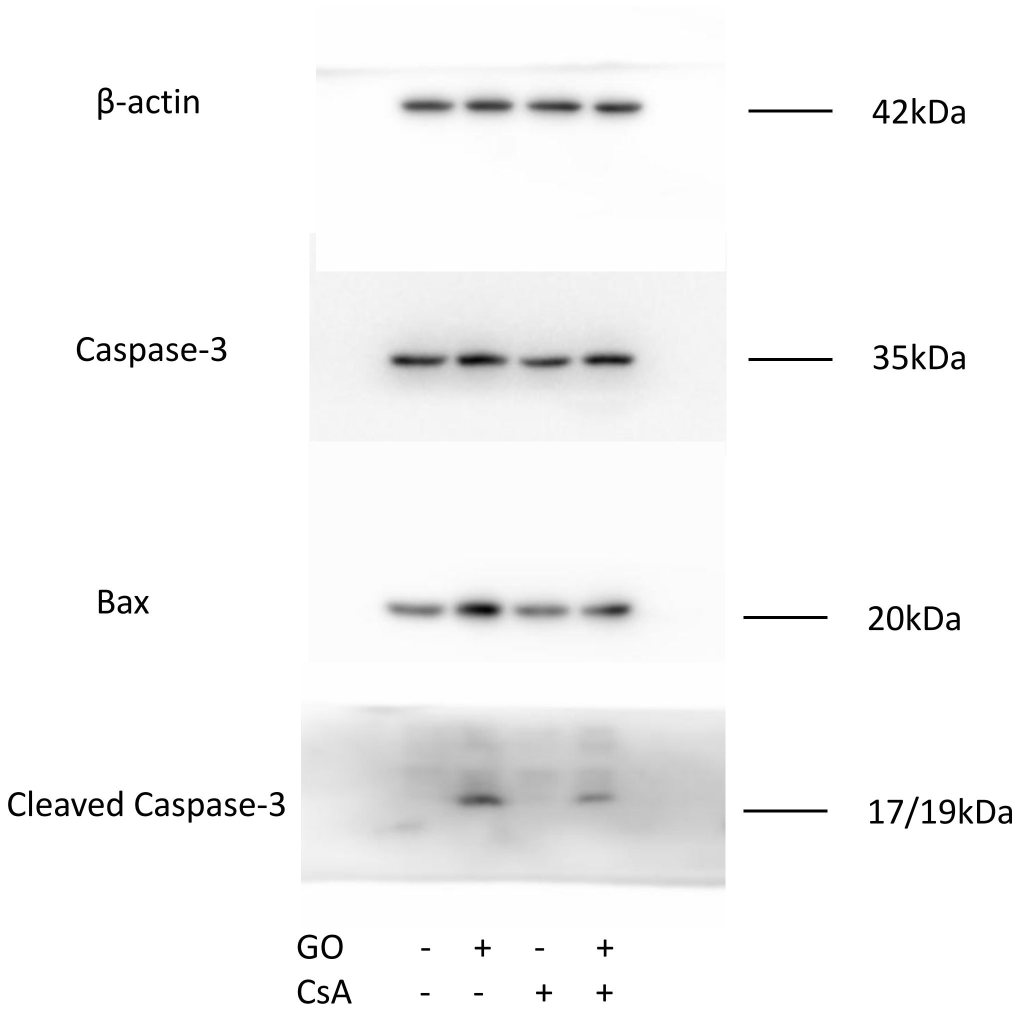

Figure. 3.
(D) Protein level of cleaved Caspase-3, Bax by Western blot.

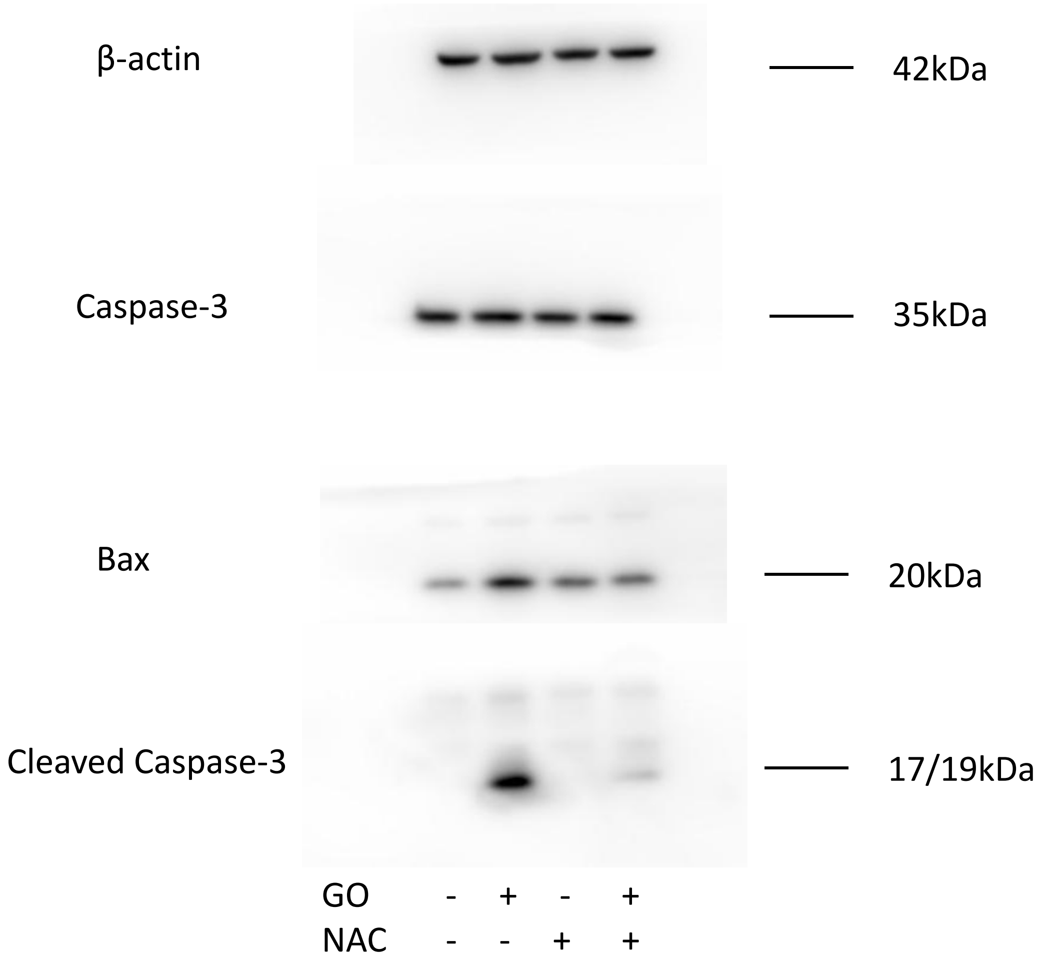

Figure. 4.
(F) Protein level of cleaved Caspase-3, Bax by Western blot.

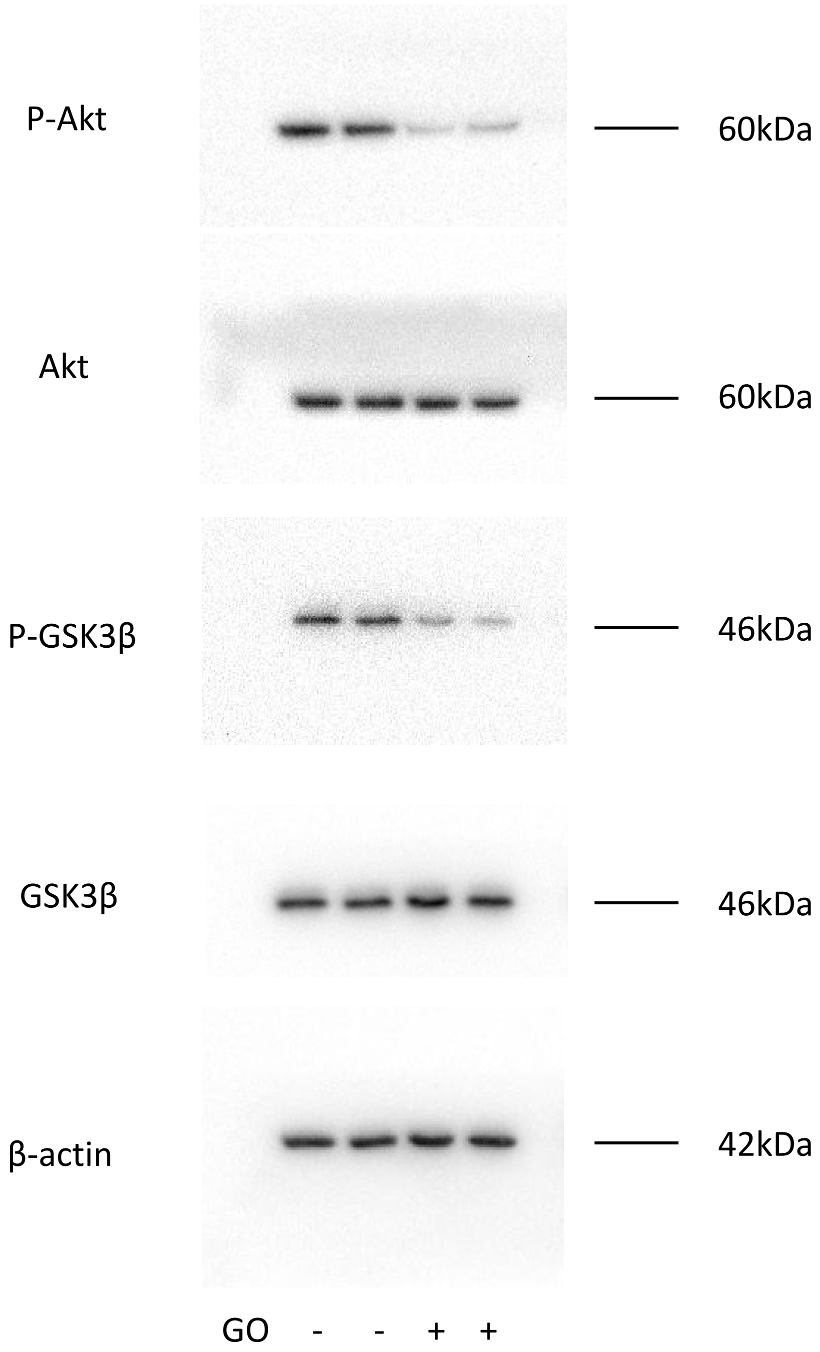

Figure. 5.
(A) Protein level of p-Akt and Akt, p-GSK3β and GSK3β in the presence of glucose oxidase by Western blot.

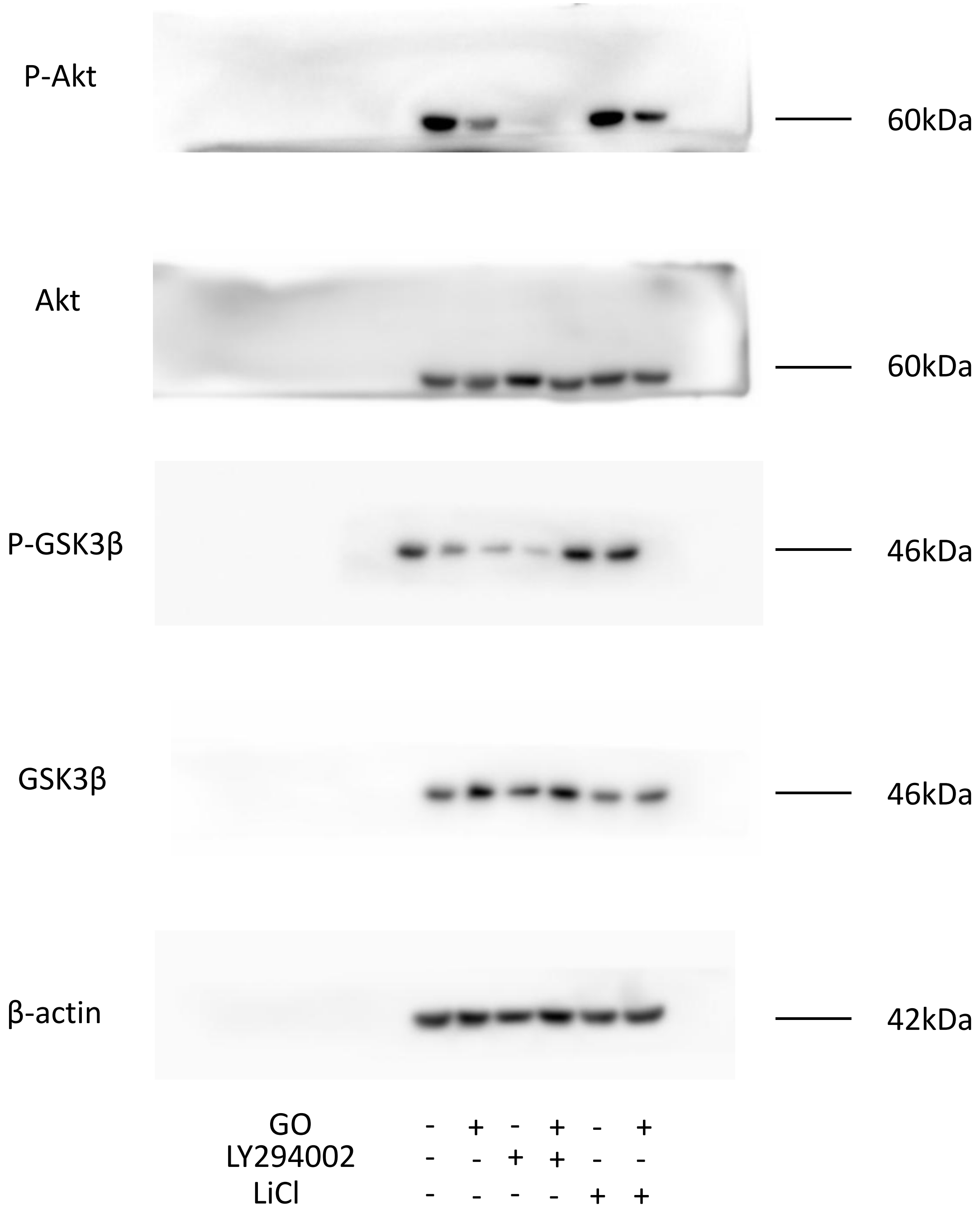

Figure. 5.
(E) Protein level of p-Akt and Akt , p-GSK3β and GSK3β treated with (+) or without (-) LY294002 or LiCl in the presence(+) or absence (-) of glucose oxidase by Western blot.

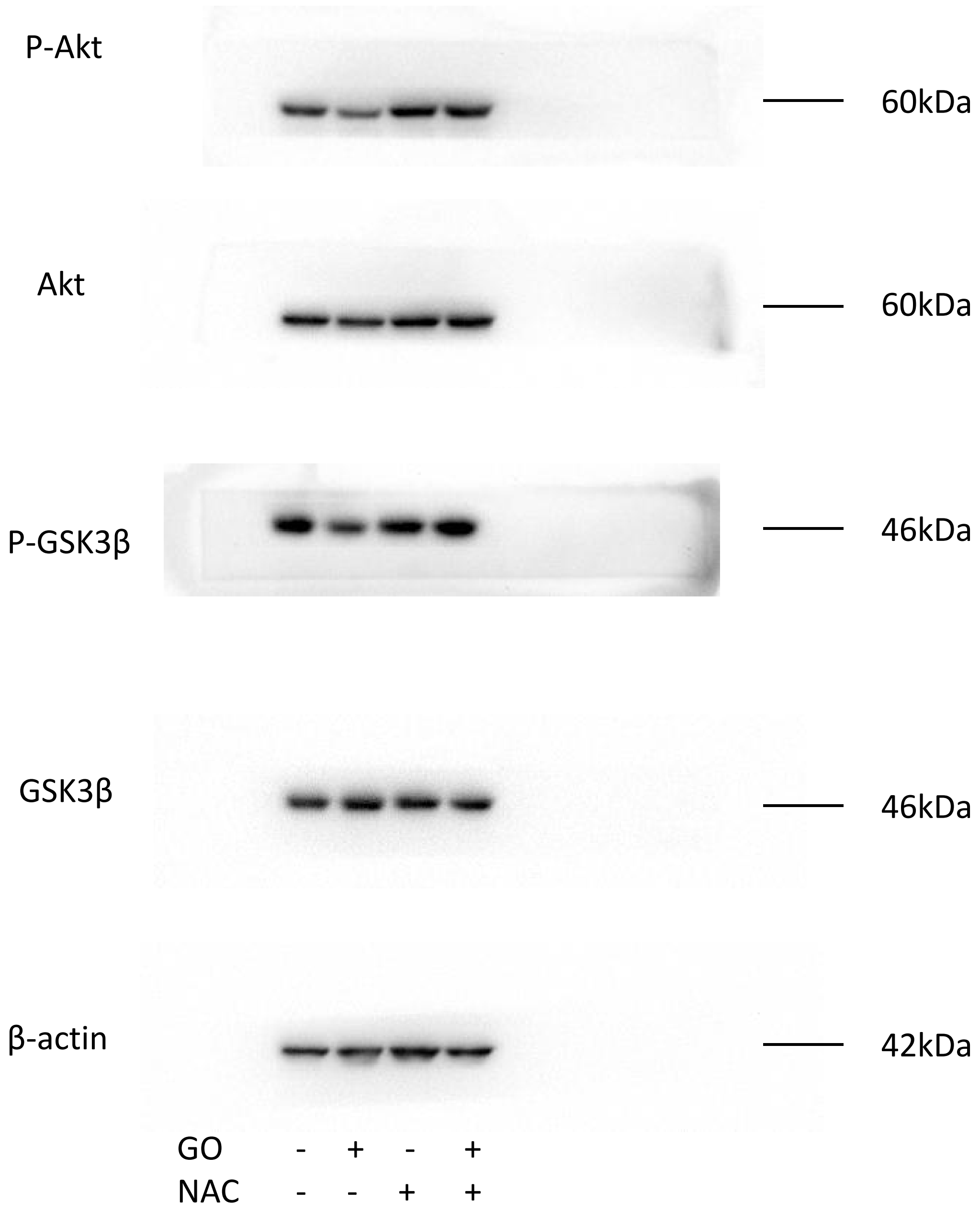

Figure. 5.
(H) Protein level of p-Akt and Akt , p-GSK3β and GSK3β treated with (+) or without (-) NAC in the presence(+) or absence (-) of glucose oxidase by Western blot.

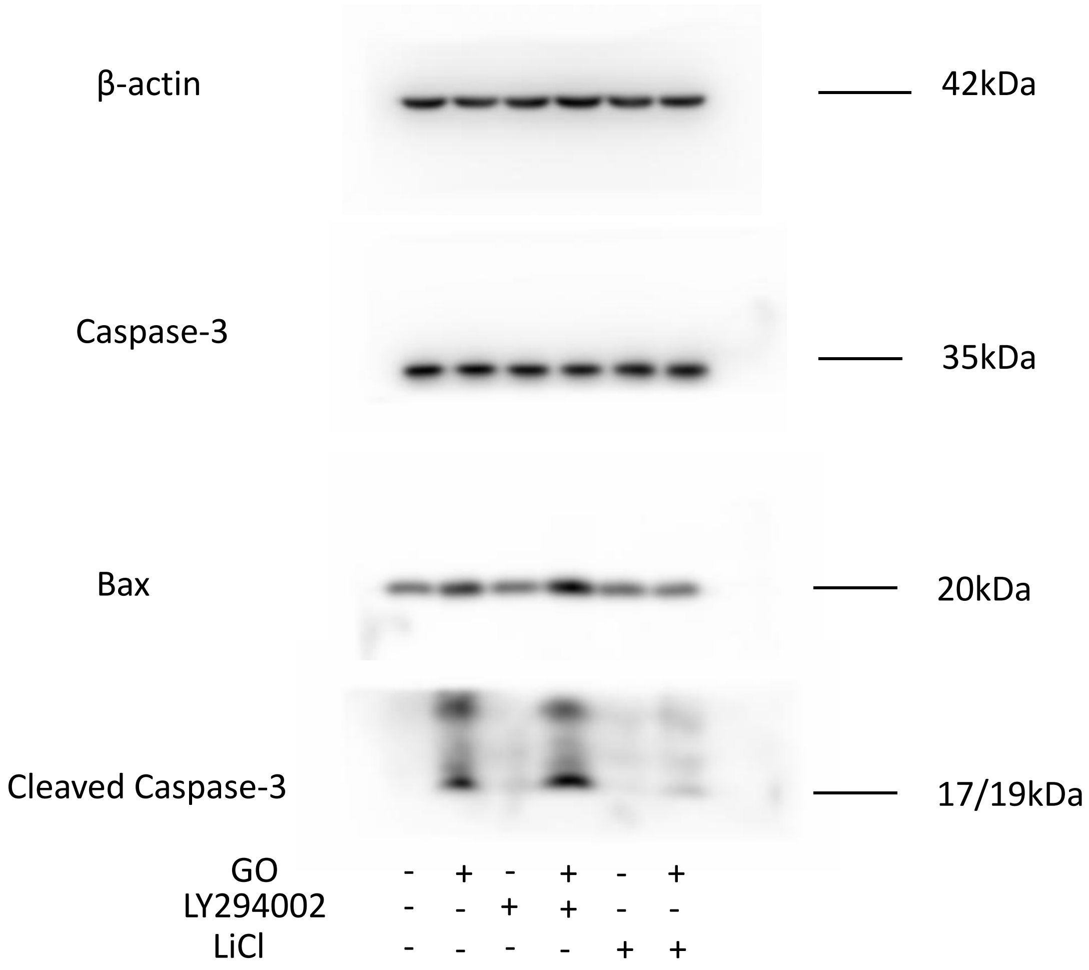

Figure. 5.
(M) Protein level of cleaved Caspase-3, Bax by Western blot.

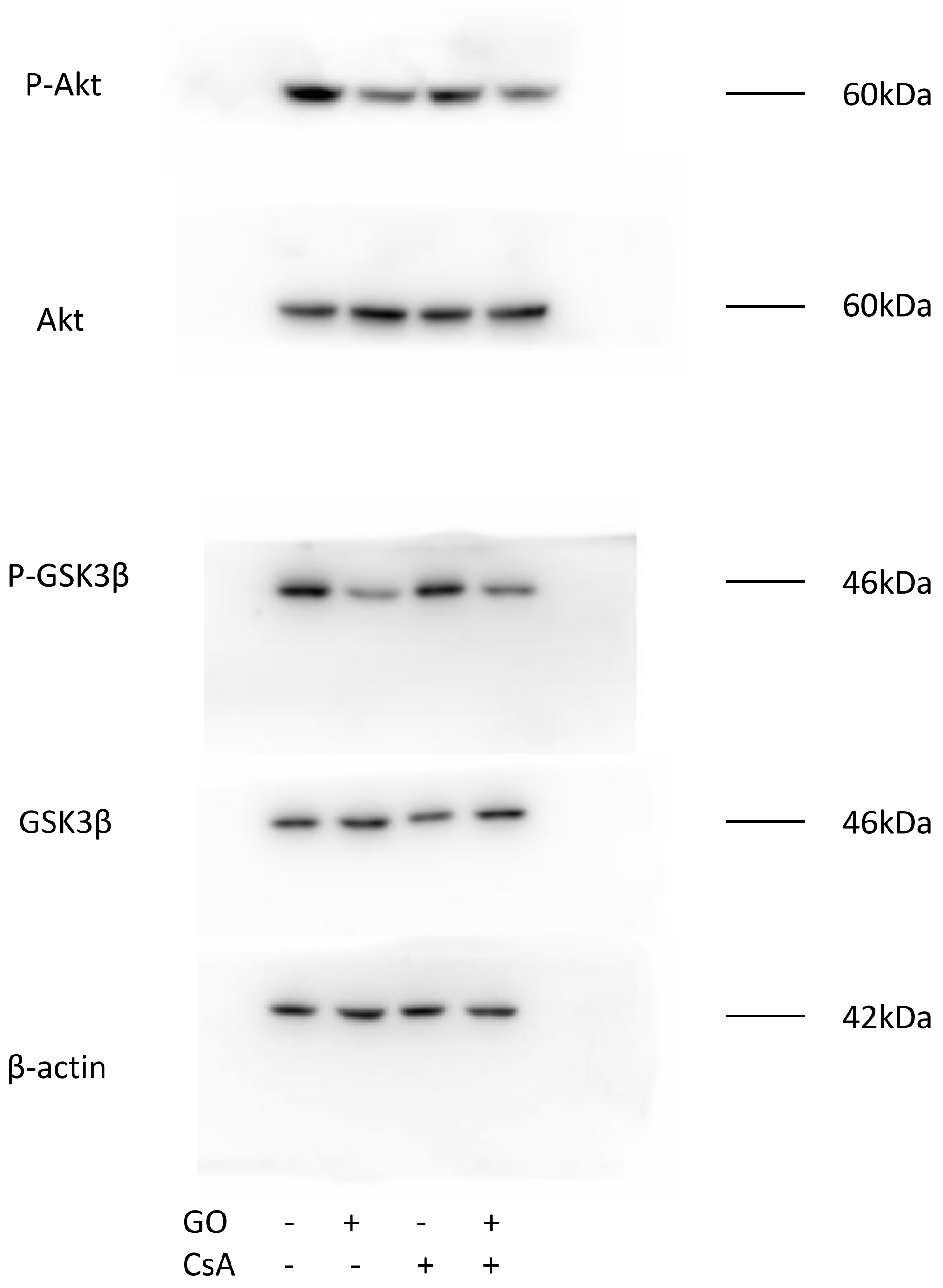

Figure. 6.
(H) Protein level of p-Akt and Akt , p-GSK3β and GSK3β treated with (+) or without (-) CsA in the presence (+) or absence (-) of glucose oxidase by Western blot.
